# Effect of Late Preharvest Deficit Irrigation on Physiological and Agronomical Responses in  ‘Regina’/Gisela 6 Sweet Cherry (*Prunus avium* L.) Cultivar

**DOI:** 10.3390/plants14040517

**Published:** 2025-02-08

**Authors:** Vanessa Huerta-Mendoza, Rocio Catalán-Paine, Iverly Romero, Jorge González-Villagra, Ricardo Tighe-Neira, Josefina Bota, Emilio Jorquera-Fontena

**Affiliations:** 1 Programa de Magister en Ciencias Agropecuarias, Departamento de Ciencias Agropecuarias y Acuícolas, Universidad Católica de Temuco, Temuco 4780000, Chile; vhuerta@uct.cl (V.H.-M.); rcatalan2022@alu.uct.cl (R.C.-P.); 2 Centro Regional Carillanca, Instituto de Investigaciones Agropecuarias, Temuco 4880000, Chile; iverly.romero@inia.cl; 3 Escuela de Agronomía, Facultad de Ciencias, Ingenieria y Tecnología, Universidad Mayor, Temuco 4801043, Chile; jorge.gonzalez@umayor.cl; 4 Departamento de Ciencias Agropecuarias y Acuícolas, Facultad de Recursos Naturales, Universidad Católica de Temuco, Temuco 4780000, Chile; rtighe@uct.cl; 5 Núcleo de Investigación en Producción Alimentaria, Facultad de Recursos Naturales, Universidad Católica de Temuco, Temuco 47780000, Chile; 6 Research Group on Plant Biology under Mediterranean Conditions, Department de Biologia, Universitat de les Illes Balears, 07122 Palma de Mallorca, Spain; j.bota@uib.es; 7 Institute of Agro-Environmental Research and Water Economy (INAGEA), 07122 Palma de Mallorca, Spain

**Keywords:** stem water potential, photosynthesis, stomatal conductance, yield, fruit quality and condition, cold storage, sorbitol

## Abstract

This study evaluated the impact of deficit irrigation during preharvest on the physiological and productive responses of ‘Regina’/Gisela 6 sweet cherry. After harvest, deficit-irrigated trees were water recovered, and physiological variables were measured. Fruit condition was evaluated after 45 days of cold storage. The experiment was carried out during the 2023–2024 season in an orchard located in La Araucanía, Chile. At 37 days after starting treatment (DAST), deficit irrigation (DI) depressed stem water potential (Ψ_s_), photosynthesis (*A^sat^*), stomatal conductance, and intercellular CO_2_ concentration with respect to controls (WI). Findings suggest that stomatal behavior was near-anisohydric on a temporal scale. Carbon partitioning into sorbitol was favored over sucrose in DI leaves, indicating improved osmoprotection. At 51 DAST, water-recovered DI trees had a Ψ_s_ equivalent to WI trees but lower *A^sat^*. Chlorophylls (SPAD) remained unaffected throughout the trial. Treatments produced similar yields, whereas DI inhibited trunk cross-sectional area growth. The DI treatment had no effect on any of the fruit quality traits other than size. Similarly, fruit condition following 45 days of cold storage was not influenced by reduced irrigation. The physiological and productive responses of ‘Regina’/Gisela 6 sweet cherry trees to preharvest deficit irrigation increased water productivity in the field.

## 1. Introduction

Sweet cherries (*Prunus avium* L.) are among the most in demand and appreciated fruits on the international market because of their flavor and health benefits. However, the sweet cherry industry faces important constraints due to water restrictions in many important sweet cherry growing regions resulting from climate change [1]. To reduce seasonal water consumption in the field, sweet cherry growers have focused on switching to efficient irrigation systems, scheduling irrigation based on soil and weather conditions and, lately, including regulated deficit irrigation practices (e.g., [2]). Deficit irrigation has been studied during the postharvest period because sweet cherry trees are usually considered susceptible to water shortages during the preharvest period [3]. However, there has not been enough research carried out on how preharvest deficit irrigation affects the physiological and productive performance of sweet cherry trees. Blanco et al. [4] reported that irrigation at 85% of crop evapotranspiration had no effect on productive and photosynthetic traits in ‘Prime Giant’ sweet cherries but depressed stem water potential and current season shoot growth. Küçükyumuk [5] documented that irrigating at 50% of field capacity from stage III of fruit development did not reduce yield in “0900 Ziraat” sweet cherries in the first year of the experiment, but trunk cross-sectional area and leaf water potential were negatively affected. Similar results were observed in the ‘Lapins’ cultivar, which experienced a 40% reduction in water supply from 60% of final fruit size to harvest, although the effect of deficit irrigation on carbon assimilation and yield seemed to be modulated by the sink effect [6]. Unaffected yield and carbon assimilation rate with reduced tree water status have been related to the anisohydric or near-anisohydric behavior of stomata in some scion/rootstock sweet cherry combinations [7]. This stomatal behavior allows plants to maintain carbohydrate production by opening their stomata for longer, despite the risk of cavitation (e.g., [8,9]). On the other hand, some studies have also revealed that physiological adaptations to water scarcity are primarily driven by the accumulation of osmotically active solutes, preserving turgor potential in the leaf cells under stress [10,11,12]. In this way, leaf and root sorbitol contents showed a remarkable increase in young sweet cherry trees subjected to water deficit [3,7].

On the other hand, postharvest techniques—primarily the control of temperature and air humidity—are crucial for the export industry due to the perishable nature of cherries [13]. The last point is particularly important for industry in the Southern Hemisphere, especially in Chile, because its exported fruit, which reached around 341,000 tons during the 2023–2024 season [14], is shipped to distant markets and is often in transit for over 40 days (Chilean industry communication). During cold storage, cherries may experience defects related to chilling, such as pedicel browning, fungal decay, pitting, and softening, which can influence the consumer purchase decision [15,16]. However, the effect of water deficit on cold-stored cherries has received little attention. Blanco et al. [17] showed that a mild water deficit (90% evapotranspiration recovered) was unable to alter the physical parameters of ‘Prime Giant’ cherries after 20 days of cold storage but produced softer fruit than well-irrigated trees.

Given that sweet cherry trees are amply cultivated in areas where the highest water demand by the fruits coincides with lower water availability, irrigation strategies must be evaluated to maintain current yields while also improving water productivity in the field. This study tests the hypothesis that late preharvest deficit irrigation decreases CO_2_ assimilation without adversely affecting yield and fruit quality and condition at harvest and following post-cold storage. A deficit irrigation treatment was applied to the late season ‘Regina’/Gisela 6 sweet cherry cultivar to assess its impact on leaf physiology, productive response, and fruit condition following 45 days of cold storage. Furthermore, the effect of postharvest rewatering on leaf gas exchange responses was evaluated.

## 2. Results

### 2.1. Soil Water Content

**Figure 1:**
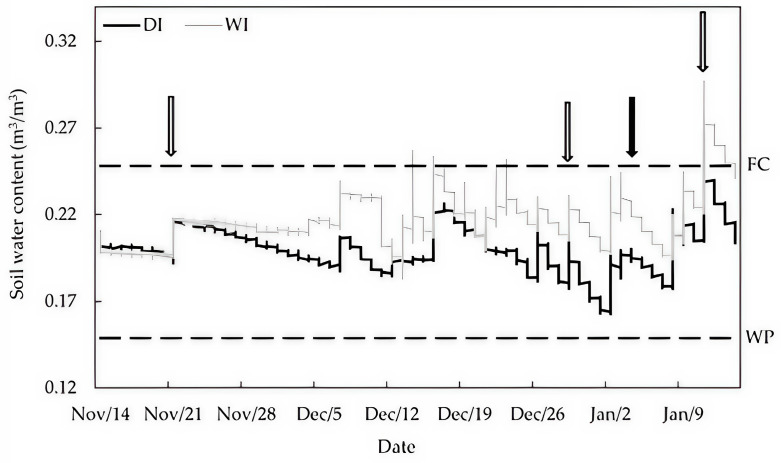
Volumetric soil water in well-irrigated (WI) and deficit-irrigated (DI) sweet cherry trees cv. ‘Regina’/Gisela 6. Field capacity (FC) and wilting point (WP) are shown in dotted lines. White arrows indicate measurement dates, while the black arrow shows the harvest time.

During the experiment, the water supplied in deficit-irrigated (DI) trees was 47% less than in well-irrigated (WI) trees (707 and 1326 m^3^ ha^−1^ for DI and WI, respectively), which was suitably detected by soil moisture sensors (Figure 1).

### 2.2. Stem Water Potential

**Figure 2:**
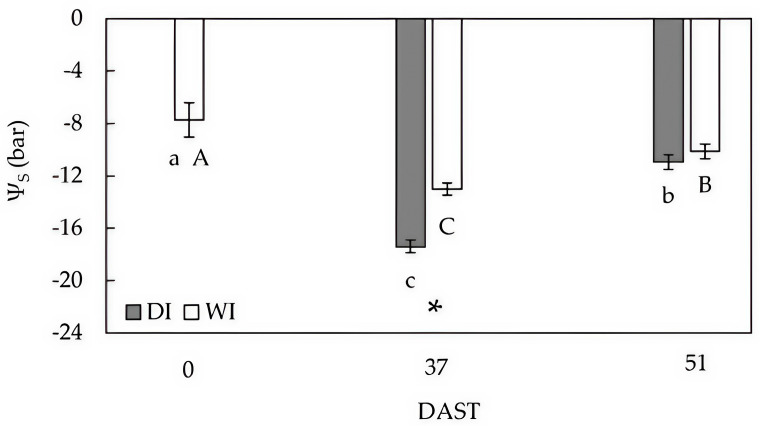
Stem water potential (Ψ_s_) in well-irrigated (WI) and deficit-irrigated (DI) sweet cherry cv. ‘Regina’/Gisela 6. DI trees were irrigated at 100% ETc for three days prior to measurements being performed at 51 DAST. Means and standard deviation are shown. Statistical differences in WI and DI at different DAST are indicated by capital and lowercase letters, respectively (Tukey test, *p* ≤ 0.05). The symbol * represents significant differences between treatments (Student *t*-test, *p* ≤ 0.05).

At 37 days after starting deficit irrigation treatment (DAST), DI had 34% lower stem water potential (Ψ_s_) than WI; however, both treatments showed a significant drop in water status with respect to values observed at 0 DAST, decreasing by around 125% and 68% for DI and WI, respectively (Figure 2).

At 51 DAST (seven days after harvest), when DI trees experienced three days of rewatering, Ψ_s_ values were similar between treatments and improved with respect to 37 DAST by 37% and 22% in DI and WI, respectively (Figure 2). Differences in Ψ_s_ measured at 0 and at 51 DAST reached 34% and 27% for DI and WI, respectively (Figure 2).

### 2.3. Leaf Gas Exchange Variables and Chlorophyll Index

**Figure 3:**
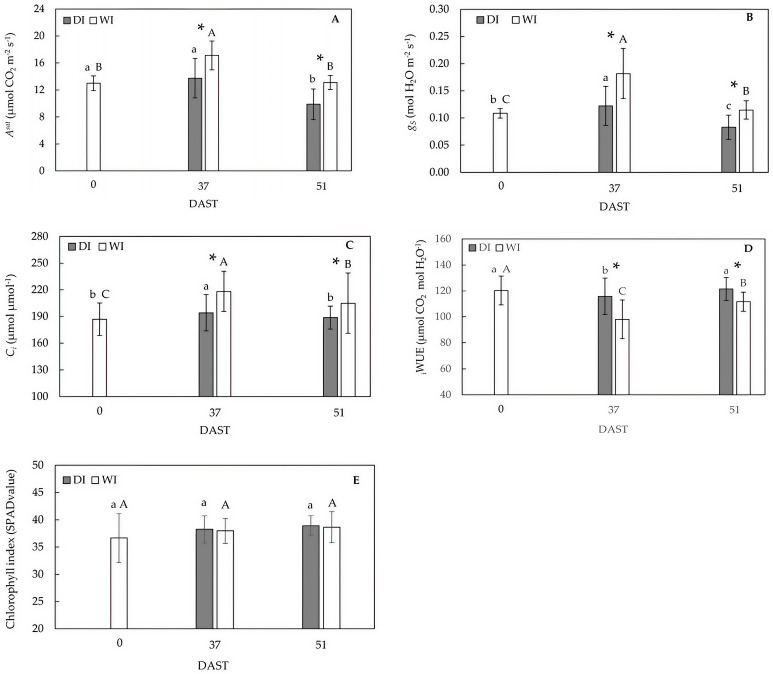
CO_2_ assimilation rate (*A^sat^*, (**A**)), stomatal conductance (*g_s_* (**B**)), intercellular CO_2_ concentration (*C_i_*, (**C**)), intrinsic water use efficiency (_i_WUE, (**D**)) and chlorophyll index (**E**) in well-irrigated (WI) and deficit-irrigated (DI) sweet cherry cv. ‘Regina’/Gisela 6. DI trees were irrigated at 100% ETc for three days prior to measurements at 51 DAST. Means and standard deviation are shown. Statistical differences in WI and DI at different DAST are indicated by capital and lowercase letters, respectively (two-to-two Dwass–Steel–Critchlow–Fligner comparisons, *p* ≤ 0.05). The symbol * represents significant differences between treatments (Mann–Whitney U test, *p* ≤ 0.05).

At 37 DAST, the effect of deficit irrigation induced significant reductions in light-saturated photosynthesis rate (*A^sat^*) (−20%), stomatal conductance to water vapor (*g_s_*) (−33%) and intracellular CO_2_ concentration (*C_i_*) (−11%), with respect to full irrigation. As expected, the intrinsic water use efficiency, measured as the *A^sat^*/*g_s_* ratio (_i_WUE), increased by about 18% in DI trees (Figure 3).

At postharvest (51 DAST), rewatered DI trees had lower *A^sat^* (−25%), *g_s_* (−27%), and *C_i_* (−8%) values and a higher _i_WUE (9%) than WI trees (Figure 3).

When comparing measurements at 0 and 37 DAST, *A^sat^* increased by around 32% in WI trees, while it remained unaffected in DI trees. Changes in *A^sat^* were accompanied by significant increases in *g_s_* (64%) and *C_i_* (17%). Although *A^sat^* remained similar from 0 to 37 DAST, *g_s_* and *C_i_* increased by approximately 9% and 4%, respectively. From 37 to 51 DAST, *A^sat^*, *g_s_*, and *C_i_* significantly dropped, while _i_WUE increased for both treatments (Figure 3). During the trial, treatment had no effect on the chlorophyll index (as SPAD value), although there was a trend towards greater values in DI trees (Figure 3E).

### 2.4. Correlation Analysis of Leaf Physiological Variables

The Ψ_s_ was exclusively and negatively related to *g_s_* (Table 1). The *A^sat^* was positively correlated to *g_s_* and *C_i_*, and negatively correlated to _i_WUE, whereas *g_s_* and *C_i_* showed a significant and positive correlation between them. *C_i_* was negatively correlated to _i_WUE. No significant correlation was observed between SPAD and any other assessed variable (Table 1).

### 2.5. Leaf Soluble Sugars

Deficit-irrigated trees showed similar glucose, fructose, and total soluble sugars to control trees, whereas sorbitol increased by 29% and sucrose dropped by 61%, approximately (Table 2).

### 2.6. Yield, Water Productivity, TCSA, and Fruit Quality at Harvest

Fruit yield was similar between treatments, but it tended to be lower due to water deficit (Table 3). The water supplied in DI treatment was sufficient to produce a substantial change in water productivity in the field, which rose by around 49% (Table 3). DI trees exhibited comparable final trunk cross-sectional areas (TCSA, cm^2^) to WI trees. The difference between the initial and final TCSA values was significant between treatments, with DI exhibiting half the growth of WI (Table 3).

Fruit size decreased due to the effect of deficit irrigation, with individual weight and caliber being 7% and 3% less, respectively. There was a marginal increase in fruit firmness in DI trees, while total soluble solids (TSS) and titratable acidity (TA) did not show substantial variations with treatment (Table 4).

### 2.7. Fruit Condition After 45 Days of Cold Storage

Irrigation treatments did not induce changes in any parameters of fruit condition after 45 days of cold storage; however, weight loss was marginally higher in DI than in WI (Table 5).

## 3. Discussion

Preharvest deficit irrigation reduced the volumetric soil water content, resulting in a significant decrease in Ψ_s_ in deficit-irrigated trees at 37 DAST (Figure 1 and Figure 2). Lower Ψ_s_ resulted in decreased *A^sat^*, *g_s_*, and *C_i_* (Figure 3), indicating that a lack of soil water depressed gas exchange activity in DI leaves as expected. These results agree with those reported by Marsal et al. [18] and Blanco et al. [4], who showed that lower Ψ_s_ caused by deficit irrigation reduced photosynthesis in ‘New Star’ and ‘Prime Giant’ sweet cherry trees. Nonetheless, the extent to which photosynthesis is reduced in sweet cherry leaves depends on the severity and/or duration of water deficit in interaction with crop phenology [17].

When comparing observations at 0 DAST and 37 DAST, we found that Ψ_s_ decreased regardless of treatment without a concomitant decrease in gas exchange variables. In fact, *g_s_* and *C_i_* values increased in both treatments, while *A^sat^* increased exclusively in WI. From these findings, we can suggest that stomatal behavior in cv. ‘Regina’ grafted onto Gisela 6 rootstock sweet cherry was near-anisohydric on a temporal scale, maintaining similar or increased CO_2_ assimilation depending on the magnitude of the irrigation-associated decrease in Ψ_s_. Recent research has demonstrated that anisohydric or isohydric behavior of young cherry trees is determined by the scion/rootstock combination and is mediated by changes in sorbitol content in leaves and roots [7]. However, it is difficult and often incorrect to categorize cultivars as strictly iso/anisohydric [19,20] because the severity of water deficit modulates the iso/anisohydric nature of plants [21]. Regarding leaf soluble sugars, we found that the pool of leaf soluble sugars did not differ between treatments, with sorbitol and fructose being the major forms of assimilated carbon at 37 DAST (Table 2). However, carbon partitioning into sorbitol was favored over sucrose in DI trees, aligning with the observations of Ranney et al. [22] and Centritto [3] in sweet cherry seedlings subjected to water deficit. Similar results were observed in young ‘Colt’ and ‘Lapins/Colt’ genotypes classified as strictly anisohydric [7]. In the same study, the sorbitol accumulation in leaves was related to high _i_WUE in concordance with the present results (*r* = 0.5; *p* = 0.018). Osmotic adjustment induced by sorbitol accumulation in leaves has been related to drought stress tolerance in several plant species and may contribute to the maintenance of open stomata at lower water potentials [23,24,25].

At 51 DAST, harvested DI trees exhibited similar Ψ_s_ to harvested WI trees as a result of three days of rewatering (Figure 1 and Figure 2), indicating that deficit irrigation sustained for 47 days was not severe enough to negatively affect the trees’ ability to transport water through the vascular system. However, *A^sat^* in rewatered DI trees did not fully recover due to a drop in *C_i_* caused by stomatal closure (Figure 3). Depressed photosynthesis after recovery by rewatering has also been associated with reduced mesophyll conductance in Mediterranean plants [26].

Measurements of gas exchange at 37 DAST and 51 DAST may reveal the effect of fruit carbon demand on photosynthesis rate. Thus, the presence of growing fruit seemed to be a significant driver for higher carbon assimilation rates as *A^sat^* decreased in harvested trees with improved Ψ_s_ (Figure 2 and Figure 3). Seasonal fluctuations in *A^sat^* have previously been documented for sweet cherry and have been linked to crop load down- or up-regulating photosynthesis rates over time [27].

The significant correlations observed among gas exchange variables *A^sat^*, *g_s_*, and *C_i_* (Table 1) suggest that losses in photosynthetic capacity were not caused by metabolic impairment but rather by increased diffusional constraints of the CO_2_ assimilation rate [28,29]. These results are consistent with the lack of a significant effect of treatment on SPAD values throughout the trial (Figure 3E), showing that chlorophyll [30,31] and leaf nitrogen [32] did not influence photosynthesis response.

The effect of deficit irrigation on _i_WUE has been widely reported in several fruit crops, including sweet cherry [4,33,34]. Thus, to the extent that water supply is diminished, _i_WUE increases, reflecting an improved carbon assimilation per unit of transpired water (e.g., [35]). These results are consistent with near-anisohydric behavior, as species that close their stomata later during drought are likely to be less carbon-limited and maintain higher assimilation for the same stomatal closure.

In our study, DI produced equivalent yields but lower TCSA growth than WI (Table 3), suggesting that the higher amount of assimilated carbon in control trees might be allocated to vegetative growth. Similar TCSA results were reported in three-year-old ‘Lapins’ sweet cherry trees subjected to water deficit, confirming that water constrains exert a significant influence on vegetative growth [36].

Because deficit irrigation was applied during the third phase of fruit development, the water limitation, combined with the reduced carbon assimilation, most likely limited cell enlargement in DI fruit, reducing its size (Table 4). In the sweet cherry industry, a higher market price is paid for larger fruits [37]. However, both treatments produced cherries categorized in the same size grade according to Chinese market standards (the main consumer of cherries worldwide); therefore, they could have similar crop value (industry communication). Smaller cherries did not result in sweeter fruits because total soluble sugars and titratable acidity remained unaffected by treatments (Table 4). Our findings are consistent with those of Blanco et al. [17] in their first year of experimentation with cv. ‘Prime Giant’ and those of Jorquera-Fontena et al. [6] with the cv. Lapins/Colt cultivar.

Irrigation treatments did not show differences in any fruit condition variable after 45 days of cold storage. However, the increase in fruit firmness with respect to values observed at harvest was noteworthy (Table 5). A similar trend was recently documented by Bustamante et al. [38] in ‘Regina’ fruits with means of 356 g mm^−1^ and 493 g mm^−1^ at harvest and after 30 days of cold storage, respectively. Einhorn et al. [39] and González-Villagra et al. [40] found a slight trend of increasing and decreasing cherry fruit firmness after cold storage, respectively; however, the storage conditions and methodologies used in the preceding studies differed from those used here. Water loss during storage was negligible (less than 1%); therefore, the similarities seen in fruit condition traits cannot be attributable to differences in storage atmosphere. Our pitting results were lower than those reported by Gonzalez et al. [41] for the ‘Regina’ cultivar. Although pitting appears to be influenced by multiple factors [42], a positive relationship between fruit firmness and pitting resistance in sweet cherry has been demonstrated previously [43]. The treatments did not influence the brow pedicel levels. However, the founded percentage of brow pedicel was lower than that reported by Einhorn et al. [39] in ‘Sweetheart’ cherries after 4 weeks of cold storage at 0 °C. In contrast to our cracking observations, Blanco et al. [34] documented that deficit irrigation had a substantial effect on this feature in ‘Prime Giant’ cherries. This difference was most likely attributable to genotype influence, given that the ‘Regina’ cultivar is known for its low susceptibility to fruit cracking [44]. In general, decay and the presence of fungi were low regardless of the treatment, although some reports have indicated that full irrigation increases rot incidence (e.g., [45]).

## 4. Materials and Methods

### 4.1. Experimental Site, Plant Material, and Treatments

The experiment was carried out in a commercial orchard in Lumaco, Araucanía Region, Chile (38°6′ N; 72°51′ W) during the 2023–2024 season. The site is characterized by warm, dry climate conditions from November to March. The soil is classified as an Ultisol with a sandy clay loam texture (46% sand, 26% lime, and 28% clay).

The plant material corresponded to 7-year-old sweet cherry trees (cv. ‘Regina’) grafted onto Gisela 6 rootstock, established at a spacing of 4 m × 1 m in two north–south oriented rows and trained in a tall spindle axis system. For the experiment, 48 trees were selected based on similar vigor vis-à-vis the trunk cross-sectional area. Pest control was carried out following the technical recommendations of the fruit export industry. Preharvest fertilization requirements were satisfied via fertigation prior to applying the deficit irrigation treatment.

For the experiment, two irrigation treatments were applied: 1) well-irrigated trees (WI), irrigated at 100% of estimated crop evapotranspiration (ET_C_), and 2) deficit-irrigated trees (DI), irrigated at 55% ET_C_ (in a similar magnitude to that reported by Blanco et al. [4] and Jorquera-Fontena et al. [6]). The DI was applied from the third phase of fruit growth (BBCH 76, 60% of final fruit size), corresponding to 42 days after full bloom (21 November 2023) to 4 days after harvest (8 January 2024), the moment when trees were water-recovered at 100% ET_C_.

**Figure 4:**
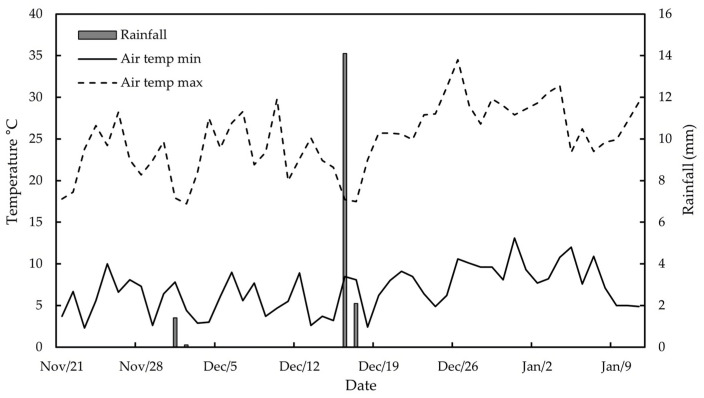
Maximum and minimum air temperatures, and accumulated precipitation during the experiment.

The ET_C_ was calculated following FAO 56, with crop coefficients obtained from the literature and reference evapotranspiration from the Gaby Ranquilco-INIA automatic weather station located 4 km from the experimental site (https://agrometeorologia.cl). Plants were irrigated with two drip lines on each row, with emitters spaced at 70 cm intervals along the lines. The emitter flow rate was 4.5 L/h for WI, which was then adjusted to 2.5 L/h for DI. Depending on demand, a two- or three-day irrigation frequency was used. During the experiment, three precipitation events were recorded, accumulating 17.7 mm. Rainfall and mean temperatures are shown in Figure 4.

### 4.2. Measurements

#### 4.2.1. Soil Water Content

Volumetric soil water content was recorded throughout the season using a Hobo soil moisture/temperature sensor model RXW-T11-922 (Hobo, Bourne, MA, USA) installed at a 30 cm depth and positioned at 50 cm from four representative trees in each treatment [46].

#### 4.2.2. Stem Water Potential

The Ψ_s_ was measured at 0, 37, and 51 DAST. Days 37 and 51 corresponded to 7 days before and after harvest, respectively, with the final day having three days of water-recovered trees. Measurements were performed using a pressure chamber (PMS Model 600, Albany, OR, USA) on enclosed leaves with an opaque plastic bag for 1 h as suggested by Choné et al. [47]. For the measurements, fully expanded mature leaves from the mid-portion of the tree were chosen at random from the mid-position of sun-exposed two-year-old branches. Measurements were taken from 12:00 to 13:00 h. A total of 22 leaves were measured each day.

#### 4.2.3. Leaf Gas Exchange

Gas exchange variables were also determined at 0, 37, and 51 DAST on sun-exposed, randomly selected leaves placed in the same above-described position of the tree. Measurements were performed between 10:00 and 16:00 h using a portable gas exchange system LI-6800 (LI-COR, Lincoln, NE, USA) equipped with a leaf chamber of 4 cm^2^. Conditions in the leaf chamber were adjusted to a photosynthetic photon flux density of 1500 µmol m^2^ s^−1^, a temperature of 22 °C, a CO_2_ concentration of 400 µmol CO_2_ mol air^−1^, and a relative humidity of 60%. Gas exchange variables were recorded upon reaching a steady state (≈10 min). From the gas exchange measurements, *A^sat^*, *g_s_* and *C_i_* were determined. Subsequently, _i_WUE was calculated. A total of 22 leaves were measured each day.

#### 4.2.4. Chlorophyll Index

After gas exchange measurements, the chlorophyll index, expressed as SPAD values, was assessed, considering Hoel and Solhaug’s [48] recommendations. An MC-100 Chlorophyll Concentration Meter (Apogee Instruments, Logan, UT, USA) was used for determinations. Using the same branch and position described in Section 4.2.2 and Section 4.2.3, leaves were randomly selected to perform two readings per leaf, which were then averaged.

#### 4.2.5. Leaf Soluble Sugars

At 37 DAST and immediately after determining gas exchange variables, leaves were detached and rapidly placed on liquid nitrogen for further analysis. In the laboratory, the extraction and analysis of leaf soluble carbohydrates were carried out following the protocol described by Usenik et al. [49]. The extracts were analyzed using an HPLC-LC 2050C system (Shimadzu Corp., Kyoto, Japan) with a RID-20A refractive index detector. A Hi-Plex Pb 300 × 7.7 mm column operated at 65 °C with HPLC-grade water used for separation. The injection volume was 20 μL, the flow rate was 0.6 mL/min, and the run time was 30 min. Quantification of the identified compounds including sucrose, fructose, glucose, and sorbitol was based on peak area expressed in mg/g of fresh weight.

#### 4.2.6. Yield, Water Productivity, and Fruit Quality at Harvest

For yield determination, 18 trees of each treatment were harvested, and then fruits were weighed using a field balance. Water productivity was calculated as the ratio between fruit yield (kg ha^−1^) and total water applied (m^3^ ha^−1^) from the beginning of the irrigation season to the moment when DI trees returned to 100% ET_C_. At harvest, 1 kg of fruit sample was randomly taken per tree and stored in a cooler for further analysis. In the laboratory, samples were stored at 4 °C. Subsequently, 3 subsample fruits per sample were selected at random (54 fruits per treatment) to determine the fresh weight using an analytical balance. Then, subsamples were crushed in a mortar, and the juice was analyzed for total soluble solids (°Brix) and titratable acidity (% citric acid) using an Atago Pocket PAL-BX/ACID16 (Atago, Tokio, Japan). The rest of the samples were stored at 0 °C and 90% RH until the next day. After this period, 10 subsample fruits (180 per treatment) were acclimated to 15 °C for 1h to determine their size and fruit firmness using a portable FirmPro (HappyAgro, Santiago, Chile).

#### 4.2.7. Trunk Cross-Sectional Area

At the end of the DI treatment (51 DAST), trunk cross-sectional area (TCSA) was determined for each tree per treatment to be compared with the TCSA values previously obtained at 0 DAST. The TCSA was calculated from the trunk perimeter measured at 20 cm from the graft union.

#### 4.2.8. Fruit Condition After 45 Days of Cold Storage

From the samples taken at harvest, about 500 g subsamples per tree were collected at random and then packed in view-fresh low-density polyethylene plastic bags for modified atmosphere (5–6% CO_2_; 16–17% O_2_) to evaluate the fruit condition 45 days after storage at 0 °C and 90% RH in the postharvest chamber [50]. After the cold storage period, subsamples were acclimatized at 15° C for 1 h and then fruits were weighed to determine weight loss. Then, 10 fruits were randomly selected per bag (180 per treatment) to determine firmness using a FirmPro tester (HappyAgro, Santiago, Chile). The remaining fruits were used to evaluate the following fruit condition parameters: orange peel, pitting, cracking, decay, fungi, internal browning, and brown pedicel expressed as percentages based on mass-based determination [51].

### 4.3. Experimental Design and Data Analysis

Irrigation treatments were applied randomly to six groups of eight trees, with each group randomly replicated. The trees were separated by two buffer trees and arranged in two parallel rows in the orchard. For the analysis, each tree was treated as an experimental unit following a randomized experimental design. The data analysis was performed using the JAMOVI version 2.3.28 statistical program. The data were first submitted to normality and equality of variances analyses using the Shapiro–Wilk and Levene tests, respectively. To evaluate the effect of the treatment at 37 and 51 DAST, a corresponding Student *t*-test or Mann–Whitney U test was performed (*p* ≤ 0.05). Repeated measurements, such as Ψ_s_, gas exchange variables, and chlorophyll index, were analyzed over time using Fisher’s or Kruskal–Wallis analysis of variance (ANOVA), and mean values were separated using Tukey’s or Dwass–Steel–Critchlow–Fligner tests (*p* ≤ 0.05), as appropriate. Differences in leaf soluble sugars, yield, TCSA, and fruit quality and condition variables were determined using Student’s *t*-test (*p* ≤ 0.05). Because of differences in sample sizes, postharvest variables were compared as transformed averages using the equation $arcsine\sqrt{x}$ [52].

## 5. Conclusions

Deficit irrigation decreased the water status and gas exchange capacity of ‘Regina’/Gisela 6 sweet cherry trees without affecting yield, fruit quality, or fruit condition at harvest and after 45 days of cold storage. These outcomes could be mediated by enhanced leaf osmoprotection, which allows stomata to remain open even at lower water potentials. Our findings also demonstrated that trees with less water prioritized fruit production over vegetative growth. The physiological and productive responses of ‘Regina’/Gisela 6 trees to preharvest deficit irrigation allowed increased water productivity in the field. Because water limitation is expected to be intensified by global climate change, further research is needed to improve our knowledge about the impact of water scarcity on different sweet cherry cultivars during the preharvest period.

## Figures and Tables

**Table 1:** Person’s correlation coefficients among evaluated leaf traits variables in ‘Regina’/Gisela 6 sweet cherry.

	Ψ_s_	*A^sat^*	*g_s_*	*C_i_*	_i_WUE
*A^sat^*	−0.264				
*g_s_*	−0.278 *	0.905 ***			
*C*_i_	−0.185	0.534 ***	0.769 ***		
_i_WUE	0.22	−0.648 ***	−0.862 ***	−0.962 ***	
SPAD	−0.194	0.021	0.023	0.028	0.036

Symbols * and *** represent statistical significance at *p* ≤ 0.05, and *p* ≤ 0.001, respectively.

**Table 2:** Leaf soluble sugars in well-irrigated (WI) and deficit-irrigated (DI) sweet cherry cv. ‘Regina’/Gisela 6 at 37 DAST. Means, standard errors, and statistical significance (Student *t*-test) at *p* ≤ 0.05 are shown.

Variable	Treatment		Significance
	WI	DI	*p*-Value
Sucrose (mg g^−1^ FW)	1.11 ± 0.28	0.43 ± 0.13	0.039
Glucose (mg g^−1^ FW)	9.36 ± 0.49	8.90 ± 0.51	0.478
Fructose (mg g^−1^ FW)	15.07 ± 0.74	15.24 ± 1.21	0.906
Sorbitol (mg g^−1^ FW)	18.75 ± 1.26	24.15 ± 1.48	0.011
Total sugars (mg g^−1^ FW)	44.29 ± 1.76	48.72 ± 2.59	0.171

**Table 3:** Yield, water productivity and trunk cross-sectional area (TCSA) in well-irrigated (WI) and deficit-irrigated (DI) sweet cherry cv. ‘Regina’/Gisela 6 subjected to different irrigation doses. Means, standard errors, and statistical significance (Student *t*-test) at *p* ≤ 0.05 are shown.

Variable	Treatment		Significance
	WI	DI	*p*-Value
Yield (kg tree^−1^)	7.77 ± 0.53	6.56 ± 0.40	0.076
Water productivity (kg m^−3^)	13.60 ± 0.92	20.29 ± 1.22	<0.001
Initial TCSA (cm^2^)	47.71 ± 2.65	45.95 ± 1.93	0.595
Final TCSA (cm^2^)	55.54 ± 3.41	49.62 ± 1.92	0.139
TCSA variation (%)	16.45 ± 3.05	8.53 ± 2.26	0.017

**Table 4:** Fruit weight, total soluble solids (TSS), titratable acidity (TA), fruit caliber, and firmness in sweet cherry cv. ‘Regina’/Gisela 6 subjected to different irrigation doses. Means, standard errors, and statistical significance (Student *t*-test) at *p* ≤ 0.05 are shown.

Variable	Treatment		Significance
	WI	DI	*p*-Value
Fruit weight (g)	9.69 ± 0.21	8.97 ± 0.26	0.035
TSS (°Brix)	21.77 ± 0.33	21.66 ± 0.31	0.893
TA (% citric acid)	0.70 ± 0.01	0.71 ± 0.02	0.990
Fruit caliber (mm)	25.58 ± 0.14	24.92 ± 0.17	0.002
Firmness (g mm^−1^)	287.03 ± 4.25	297.81 ± 4.32	0.077

**Table 5:** Variables of fruit condition after 45 days of cold storage in well-irrigated (WI) and deficit-irrigated (DI) sweet cherry cv. ‘Regina’/Gisela 6. Means, standard errors, and statistical significance (Student *t*-test) at *p* ≤ 0.05 are shown.

Variable	Treatment		Significance
	WI	DI	*p*-Value
Firmness (gf/mm)	405.30 ± 6.27	400.85 ± 7.17	0.637
Weight loss (%)	0.47 ± 0.04	0.60± 0.06	0.058
Orange peel (%)	18.20 ± 1.28	18.52 ± 0.97	0.679
Pitting (%)	22.91 ± 2.07	20.25 ± 2.74	0.516
Cracking (%)	1.03 ± 0.43	1.92 ± 0.60	0.489
Decay (%)	0.21 ± 0.15	0.20 ± 0.14	0.970
Fungi (%)	1.59 ± 0.52	1.43 ± 0.56	0.754
Internal browning (%)	7.41 ± 2.01	9.26 ± 2.01	0.741
Brown pedicel (%)	49.07 ± 4.56	50.00 ± 5.56	0.577

## Data Availability

The raw data supporting the conclusions of this article will be made available by the authors on request. The data are not publicly available due to privacy.
